# Supplementary material for: Physical health of care-experienced young children in high-income countries: a scoping review protocol
Source: BMJ Open. 2022 Sep 7;12(9):e063648. doi: 10.1136/bmjopen-2022-063648 (PMC9454045; doi:10.1136/bmjopen-2022-063648)
Supplement: Supplementary data [file bmjopen-2022-063648supp001.pdf]

A. KEYWORDS

Care-experienced children

“care experienced child” OR cec OR "looked after child*” OR lac OR lacyp OR “out of home care” OR “foster* child*” OR “foster* care” OR “foster placement” OR “kinship care*” OR “substitute care” OR “kith and kin care” OR “friends and family care” OR “adopted child*” OR orphan* OR “residential care” OR “group home*” OR “child* home” OR “care home” OR “local authority care” OR “corporate parent” OR “child* protect*” OR “public care” OR “institution* care*” OR “supported living” OR “social care” OR “care placement” OR “child* welfare” OR “protective custody” OR “state custody”

Health

health OR healthcare OR medicat* OR condition* OR patholog* OR feeding OR growth OR thriv* OR stature OR stunting OR immuni* OR infect* OR symptom* OR disease* OR illness* OR “well-being” OR wellbeing OR neurodevelop* OR treatment* OR prescri* OR hospital*

Age

infan* OR pre-school OR preschool OR baby OR neonat* OR “under 6” OR “under six” OR “early intervention” OR birth OR “young child*” OR toddler

B. SEARCH STRATEGIES

MEDLINE via Ovid

Note: Lines beginning with “exp” instruct the database to explode the subject heading to include subsidiary terms.

1	exp Child, Foster/
2	exp Child, Adopted/
3	exp Foster Home Care/
4	(“care experienced child*” OR “looked after child*” OR lac OR “foster* child*” OR “adop* child*” OR orphan*).ab,ti.
5	(("out of home" OR foster* OR "friends and family" OR "kith and kin" OR kinship OR local authorit* OR institution* OR substitute) adj1 care).ab,ti.
6	("care home" OR "group home" OR "child* home").ab,ti.
7	("care placement" OR "foster placement").ab,ti.
8	("corporate parent" OR "child* protect*" OR "public care" OR "protective custody" OR "child* welfare" OR "state custody" OR "supported accommodation" OR "supported living").ab,ti.
9	(infan* OR pre-school OR preschool OR baby OR babies OR neonat* OR "under 6" OR "under six" OR "early intervention" OR birth OR "young child*" OR toddler*).ab,ti.
10	exp Child, Preschool/
11	(health OR healthcare OR medicat* OR condition* OR patholog* OR feeding OR growth OR thriv* OR stature OR stunting OR immuni* OR infect* OR symptom* OR disease* OR illness* OR "well-being" OR wellbeing OR neurodevelop* OR treatment* OR prescri* OR hospital*).ab,ti.
12	exp Child Development/
13	1 OR 2 OR 3 OR 4 OR 5 OR 6 OR 7 OR 8
14	9 OR 10
15	11 OR 12
16	13 AND 14 AND 15

CINAHL via EBSCOHost

Note: Lines beginning with MH are subject headings/keywords. The "+" operator instructs the database to explode the subject heading to include subsidiary terms.

S1	MH(Child, Abandoned OR Child, Adopted OR Child, Foster OR Child, Institutionalized OR Foster Home Care)
S2	TX(care experienced child* OR looked after child* OR lac OR foster* child* OR adop* child* OR orphan*)
S3	TX(("out of home" OR foster* OR "friends and family" OR "kith and kin" OR kinship OR local authorit* OR institution* OR substitute) N1 care)
S4	TX("care home" OR "group home" OR "child* home")
S5	TX("care placement" OR "foster placement")
S6	TX("corporate parent" OR "child* protect*" OR "public care" OR "protective custody" OR "child* welfare" OR "state custody" OR "supported accommodation" OR "supported living")
S7	MH("Infant+" OR Child, Preschool)
S8	TX(infan* OR pre-school OR preschool OR baby OR babies OR neonat* OR "under 6" OR "under six" OR "early intervention" OR birth OR "young child*" OR toddler*)
S9	MH(Child Health OR Dental Care for Children OR "Child Health Services+")
S10	TX(health OR healthcare OR medicat* OR condition* OR patholog* OR feeding OR growth OR thriv* OR stature OR stunting OR immuni* OR infect* OR symptom* OR disease* OR illness* OR "well-being" OR wellbeing OR neurodevelop* OR treatment* OR prescri* OR hospital*)
S11	S1 OR S2 OR S3 OR S4 OR S5 OR S6
S12	S7 OR S8
S13	S9 OR S10
S14	S11 AND S12 AND S13

Web of Science Core Collection

1 TI=("care experienced child*" OR "looked after child*" OR lac OR "foster* child*" OR "adop* child*" OR orphan*) OR AB=("care experienced child*" OR "looked after child*" OR lac OR "foster* child*" OR "adop* child*" OR orphan*)

2 TI=(("out of home" OR foster* OR "friends and family" OR "kith and kin" OR kinship OR “local authorit*” OR institution* OR substitute) NEAR/1 care) OR AB=(("out of home" OR foster* OR "friends and family" OR "kith and kin" OR kinship OR “local authorit*” OR institution* OR substitute) NEAR/1 care)

3 TI=("care home" OR "group home" OR "child* home") OR AB=("care home" OR "group home" OR "child* home")

4 TI=("care placement" OR "foster placement") OR AB=("care placement" OR "foster placement")

5 TI=("corporate parent" OR "child* protect*" OR "public care" OR "protective custody" OR "child* welfare" OR "state custody" OR "supported accommodation" OR "supported living") OR AB=("corporate parent" OR "child* protect*" OR "public care" OR "protective custody" OR "child* welfare" OR "state custody" OR "supported accommodation" OR "supported living")

6 TI=(infan* OR pre-school OR preschool OR baby OR babies OR neonat* OR "under 6" OR "under six" OR "early intervention" OR birth OR "young child*" OR toddler*) OR AB=(infan* OR pre-school OR preschool OR baby OR babies OR neonat* OR "under 6" OR "under six" OR "early intervention" OR birth OR "young child*" OR toddler*)

7 TI=(health OR healthcare OR medicat* OR condition* OR patholog* OR feeding OR growth OR thriv* OR stature OR stunting OR immuni* OR infect* OR symptom* OR disease* OR illness* OR "well-being" OR wellbeing OR neurodevelop* OR treatment* OR prescri* OR hospital*) OR AB=(health OR healthcare OR medicat* OR condition* OR patholog* OR feeding OR growth OR thriv* OR stature OR stunting OR immuni* OR infect* OR symptom* OR disease* OR illness* OR "well-being" OR wellbeing OR neurodevelop* OR treatment* OR prescri* OR hospital*)

8 1 OR 2 OR 3 OR 4 OR 5

9 6 AND 7 AND 8

C. DATA CHARTING VARIABLES

Bibliographic variables

Article title, authors, year of publication, journal title.

Participant demographics and setting

Age (inc. measure of central tendency/dispersion), gender split, care placement type, country.

Study-related variables

Health outcomes studied, prevalence statistics/statistical methods used, effect sizes (where appropriate), study aims/objectives, study design, sample type(s), no. of care-experienced children in sample, no. of non-care children in control sample (where appropriate), key findings of relevance to review question.
