# Supplementary material for: Physical health of care-experienced young children in high-income countries: a scoping review protocol
Source: BMJ Open. 2022 Sep 7;12(9):e063648. doi: 10.1136/bmjopen-2022-063648 (PMC9454045; doi:10.1136/bmjopen-2022-063648)
Supplement: Author's manuscript [file bmjopen-2022-063648.draft_revisions.pdf]

BMJ Open

BMJ Open is committed to open peer review. As part of this commitment we make the peer review history of every article we publish publicly available.

When an article is published we post the peer reviewers' comments and the authors' responses online. We also post the versions of the paper that were used during peer review. These are the versions that the peer review comments apply to.

The versions of the paper that follow are the versions that were submitted during the peer review process. They are not the versions of record or the final published versions. They should not be cited or distributed as the published version of this manuscript.

BMJ Open is an open access journal and the full, final, typeset and author-corrected version of record of the manuscript is available on our site with no access controls, subscription charges or pay-per-view fees (http://bmjopen.bmj.com).

If you have any questions on BMJ Open's open peer review process please email info.bmjopen@bmj.com

BMJ Open

Physical health of care-experienced young children in high-income countries: a scoping review protocol

Journal:	*BMJ Open*
Manuscript ID	bmjopen-2022-063648
Article Type:	Protocol
Date Submitted by the Author:	06-Apr-2022
Complete List of Authors:	Bradford, Daniel ; University of Glasgow, MRC/CSO Social and Public Health Sciences Unit<br>Allik, Mirjam; University of Glasgow, MRC/CSO Social and Public Health Sciences Unit<br>McMahon, Alex; University of Glasgow, School of Medicine, Dentistry & Nursing<br>Brown, Denise ; University of Glasgow, MRC/CSO Social and Public Health Sciences Unit
Keywords:	PAEDIATRICS, GENERAL MEDICINE (see Internal Medicine), Community child health < PAEDIATRICS

SCHOLARONE™
Manuscripts

Title: Physical health of care-experienced young children in high-income countries: a scoping review protocol

Authors: D. R. R. Bradford[1], M. Allik[1], A. D. McMahon[2], and D. Brown[1]

Affiliations:

1. MRC/CSO Social and Public Health Sciences Unit, University of Glasgow, Berkeley Square, 99 Berkeley Street, Glasgow. G3 7HR.
2. School of Medicine, Dentistry & Nursing, K12 Level 8, 378 Sauchiehall Street, Glasgow Dental Hospital & School, Glasgow. G2 3JZ.

ORCiD: D. R. R. Bradford, M. Allik, A. D. McMahon, and D. Brown

Correspondence: D. R. R. Bradford - d.bradford.1@research.gla.ac.uk

Word count: 2694 (Inc. headers, tables, in-text citations. Exc. front matter, abstract, references, supplemental file.)

Keywords: care-experienced children; child health; foster care; infant health; physical health

For peer review only

ABSTRACT

Introduction

Care-experienced children have poorer health, developmental, and quality of life outcomes across the lifespan compared to children not in care. These inequities begin to manifest in the early years. The purpose of the proposed scoping review is to collate and synthesise studies of the physical health of young care-experienced children. The results of the review will help map the distribution of health outcomes, identify potential targets for intervention, and assess gaps in the literature relating to this group.

Methods and analysis

We will carry out a scoping review of the literature to identify studies of physical health outcomes in care-experienced children. Systematic literature searches will be carried out on the MEDLINE, CINAHL, and Web of Science Core Collection databases and results screened against inclusion/exclusion criteria. Studies will be included where participants are aged three months or greater and less than six years. Data elements extracted from included studies will include study objectives, health outcomes, participant demographics, care setting characteristics, and bibliographic information. The results of the review will be synthesised and reported using a critical narrative approach. Comparisons between care and non-care populations will be reported if sufficient studies are identified.

Ethics and dissemination

Data will be extracted from publicly available sources so no additional ethical approval is required. Results will be published in a peer-reviewed journal article. Further, they will be shared in summary reports and presented to local authorities, care organisations, and other relevant stakeholders that can influence healthcare policy and procedure relating to young children in care.

For peer review only

Abstract word count: 244 (exc. headers)

For peer review only

STRENGTHS AND LIMITATIONS OF THIS STUDY

- This will be the first review of the literature regarding physical health outcomes of young care-experienced children.
- The findings of this review will be of direct relevance to national and international health policy related to young children in care. Results will be shared with stakeholders that are able to influence policy and practice.
- Results will provide context and guidance for a planned future administrative data study of population-level health outcomes of children aged 2.5 years in Scotland.
- Out methodology follows validated review and reporting procedures to ensure a transparent and replicable study.
- Studies will be limited to those written in the English language only.

For peer review only

BACKGROUND

A significant number of infants require support beyond that which their biological parents can provide. To have these additional support needs met, some children come formally under the care of local authorities or social care services. This may mean receiving extra input from health and social care workers while remaining in the parental home; living in kinship care; living in foster care; being permanently adopted by non-biological parents; or living in residential care homes. The proportion of children in care varies internationally due to differing legal and cultural approaches. Examples from high-income countries include 14 per 1,000 children in Scotland,[1] 6.7 per 1,000 in England,[2] and 4.0 per 1,000 infants in Sweden.[3] These cross-sectional census figures underestimate the number of children that have ever been in care since there is a constant flux of children into and out of care. Although care-experienced children constitute a small portion of the population of children in high-income countries, these children deserve increased attention due to the additional challenges they face to achieving good health.[4] There is a clear motivation to provide support to improve health outcomes for such children.

Children with care experience have poorer outcomes in many domains of health and wellbeing compared to their peers in the non-care population.[5–9] Mental ill health is prevalent across the lifespan in this group and has been the subject of many reviews.[10–12] Similarly, developmental delay and disorder are common in care-experienced children and these topics have been reviewed previously.[12–14] The literature on physical health problems in care-experienced children is less well-established, particularly for those in the early years. But despite physical ill health problems being highly prevalent in this population,[15–18] these health outcomes have not yet been the subject of a review. Bringing together the results of studies which investigated this topic is therefore a useful exercise and is the purpose of the present work. Our results will help identify common conditions affecting children in care, highlight health conditions and inequalities that may

For peer review only

benefit from the development of specific interventions, and identify potential gaps in the literature to be addressed.

The results of studies comparing physical health outcomes in care-experienced children and children that have never been in care are less clearly polarised than those for mental health and development, where the former group are unequivocally worse off.[17,19,20] In the physical health domain there are instances where care-experienced children are observed to have a reduced disease burden, although these are typically for less severe disease. For example, asthma has been found to be less prevalent in samples of children in care than controls from the non-care population.[20,21] By contrast, Turney and Wildeman reported a significantly higher crude proportion of children aged 0—17 years in foster care with asthma.[17] However, after adjusting for birth and socioeconomic factors the difference in proportion compared to the non-care group became negligible. Fleming et al also reported no significant difference in asthma prevalence (after adjustment for birth and socioeconomic factors) in their population-level administrative health data study of school-aged children.[19] Reviewing the available literature can help to clarify such ambiguities. Of course, there is a substantial risk that reports of reduced healthcare need in children in care are due to differences in health-seeking behaviour rather than truly better health. Regardless, improved knowledge about differences in the prevalence of physical ill health between children in care and children the general population is needed to understand potential associations and/or causes.

While some differences in physical health conditions between children in care and children in the general population are conflicting or ambiguous, the subset of conditions caused by, or strongly associated with, abuse and neglect — such as bone fractures and poisonings — are evidently more common in care-experienced populations.[3,22–24] This is unsurprising since abuse and neglect are common reasons for a child becoming formally cared for.[25–27] Although these traumatic experiences have clear and well-documented consequences for socioemotional development,[28] there are also consequences for physical health.

For peer review only

Hospitalisations due to both injury and self-harm have been found to be more common in school-aged children in care than non-care peers after adjusting for a range of socioeconomic and maternal factors.[19] Neglectful parenting also has consequences for conditions where proactive health behaviours are vital. For example, dental health problems were significantly more frequent in children in care during a universal dental health screening programme in Scotland.[6] This difference remained after adjustment for socioeconomic status and echoes previous studies reporting increased dental health issues in this group.[16,18,21,29] Similarly, care-experienced children are more likely to have incomplete immunisation, or to have been fully immunised at older ages.[16,18] These examples highlight that children in care face unique challenges to their health. These challenges are compounded by factors deleterious to health that are associated with poorer socioeconomic circumstances which care-experienced children more often reside in.[6,30] Identification and mitigation of the consequences of these unique risk factors can help to improve health policy and practices relating to this group.

Infancy and childhood are a period where growth and development are highly sensitive to factors in the individual's environment. Being subject to emotional and physical adversity during this period is associated with long-term negative physical health outcomes.[31,32] If untreated or uncorrected during early childhood, some malleable impairments to health may become permanent. Strabismus (misalignment of the visual axes of the eyes) is an example of such a correctable condition; intervention to treat this condition in the early years is key to avoiding this becoming a lifelong issue.[33] Additionally, children that enter care early in life also tend to experience different pathways to those becoming cared for during middle childhood or adolescence.[34] Taken together, this suggests children with experience of formal care in the early years may exhibit a distinct cluster of healthcare needs compared to similarly-aged children that are not in care. Additionally, their healthcare needs likely differ from children that first become cared for later in life. By investigating physical health in the

For peer review only

younger care-experienced group in more detail it is possible that opportunities for early intervention can be better exploited to improve subsequent wellbeing.

Study objectives

Previous reviews and meta-analyses have looked at development,[12,13,28,35] mental health,[10,12,36] effectiveness of interventions and engagement with healthcare providers,[37–39] or focused on specific subgroups of children with care-experience (e.g., children with prenatal substance exposure,[40] young adults leaving care[41]). Based on preliminary searches of MEDLINE and the Cochrane Database of Systematic Reviews, it appears there has not been a review of physical health outcomes in young care-experienced children to date. The present review aims to fill that gap. A scoping review is an appropriate methodology for this purpose. This type of review will establish the scope and common themes within existing research into the topic and indicate which physical health conditions are frequently observed in young care-experienced children. This will be of interest to policy makers and healthcare practitioners.[42–44] Further, under-researched health conditions may be identified by comparing the results of this review with the more substantial body of literature on the physical health of young children that are not in care.

Concept definition

This review will address the literature on physical health. We have defined physical health conditions as those primarily rooted or expressed in physiology. While we recognise that developmental and psychiatric conditions can have organic and/or physiological aspects, these conditions have been previously comprehensively covered by other reviews (see above). In addition, the proposed review is restricted to children in care in low- and middle-income countries. This is because these children face notably different types of physical health conditions to those in high-income countries.[45,46]

For peer review only

METHODS AND ANALYSIS

This review has been designed in line with the Joanna Briggs Institute (JBI) guidelines for carrying out scoping reviews.[47] It is also informed by Arksey and O'Malley's original framework [42] and the additional guidance laid out by Levac, Colquhoun, and O'Brien.[43] The five steps of the review procedure of Arksey and O'Malley are detailed below. The JBI framework aligns with the Preferred Reporting Items for Systematic Reviews and Meta-analyses extension (PRISMA-ScR) statement on reporting and conduct of scoping reviews,[48] also used herein.

Patient and public involvement

There was no patient nor public involvement in this review protocol.

Stage 1: Identifying the research question

The Population, Concept, Context framework (PCC) was used to define the review question. Elements of the PCC are described in detail in in Table 1. This review intends to primarily answer the question: "Which physical health conditions have been studied or observed to affect young care-experienced children in high-income countries?" If there are sufficient relevant primary studies which compare care-experienced children to control groups in the general, non-care population then the review will also address the question: "How do these health conditions affect children in care compared to their peers in the general population?"

Population	Care-experienced children who meet all following criteria:<br>• Aged greater than or equal to three months and less than 6y<br>• Have experience of formal care settings listed in the introduction of this protocol, or similar settings<br>• Not adopted during the first three months of their life<br>• Not cared for in healthcare facilities, to avoid confounding

For peer review only

Concept	Physical health outcomes will be considered as those health conditions which are primarily rooted in or expressed through physiology. As a guide, these conditions will be those that are not listed in the International Classification of Diseases (11th Revision) [49] diagnostic manual top-level entity on *Mental, behavioural or neurodevelopmental disorders*.
Context	Included studies will be restricted to those carried out in high-income countries. This is because children in care in low- and middle-income countries face significantly different physical health issues.[45,46]

Table 1: Detailed description of the Population, Concept, and Context used a foundation for the review research questions.

Stage 2: Identifying relevant studies

Keywords and synonyms to identify 1) care-experienced children; 2) health and wellbeing; and 3) children aged greater than or equal to three months and less than six years will be combined to search the bibliographic databases. Studies of the health of care-experienced children previously known to the authors were used to identify keywords to form the basis of the search strategy for this review. Iterative pilot searches of MEDLINE were carried out to identify additional relevant keywords. This process ended when saturation was reached, and additional keywords/variants did not bring forth additional search results. Advice on keywords and search strategy was also taken from an Information Scientist within the University of Glasgow MRC/CSO Social and Public Health Sciences Unit. These keywords are detailed in an attached supplementary file. The review will search for relevant studies indexed by the MEDLINE, CINAHL, and Web of Science Core Collection (Science Citation Index [SCI-EXPANDED] and Social Sciences Citation Index [SSCI]). An example search strategy for MEDLINE (via the Ovid interface) is provided in an attached supplementary file.

For peer review only

The search strategy will be adapted appropriately for the remaining databases. Following searches, all results will be imported to Rayyan.[50] Duplicates will be removed.

Stage 3: Study selection

Search results will be screened for relevance to the Population Concept, and Context described above. More specific inclusion and exclusion criteria are described below. Screening will be carried out using Rayyan.[50] Screening will take place in two stages: title and abstract screening then full texts. All titles and abstracts will be screened by DRRB and a minimum of 20% will be screened independently by a second reviewer. If it is unclear whether an article should be included based on the details in the title/abstract and there is potential for it to be relevant then it will be progressed for full-text screening (e.g., if the study clearly includes care-experienced children but the age of participants is not immediately apparent it will be progressed to the full-text review stage). Crude inter-rater agreement will be reported along with Cohen's Kappa and Scott's Pi. If crude agreement falls below 95% in this sample of 20% of titles/abstracts then all items will be screened by at least two reviewers. During the next stage of screening all full-texts will be screened by DRRB and at least 20% will be screened by a second reviewer. Crude inter-rater agreement will be reported along with Cohen's Kappa and Scott's Pi. If crude agreement falls below 95% then all full-texts will be screened by at least two reviewers. Disagreements at any stage of screening will be resolved by discussion between the two reviewers. If consensus is not achieved, a third reviewer will make the final decision. Reasons for exclusion will be recorded and documented at each stage and reported in line as per the PRISMA-ScR guidelines.[48]

Inclusion criteria

- Included studies will consider children currently living in family care with additional formal support from social or child protection services, kinship care, foster care, residential care, as well as children living with adopted families. Studies of children

For peer review only

with previous experience of these settings will also be included, as will studies which aggregate groups of children either currently in or having previously been in care.

- Studies will be included if all children are aged greater than or equal to three months and less than six years, or if subgroup data for this group are reported.
- Study outcomes must include physical health conditions or diagnoses by a health professional, diagnostic manual, or validated measure(s). Studies which have made minor modifications to validated instruments may be included at reviewers' discretion. Studies which use parental report of a current or previous diagnosis by a clinician will be included.
- Studies will be included if they were carried out in high-income countries as defined by the World Bank.[51]
- Only peer-reviewed articles and reports will be included.

Exclusion criteria

- Studies of children living in healthcare settings will be excluded to avoid some degree of confounding.
- Studies where children than have spent less than three months from birth to entering care (i.e., neonatal adoption).
- Studies of international adoptees from low- and middle-income countries to high-income countries.
- Papers that look at only physiological markers will be excluded. For example, studies of expression of hormones, such as salivary cortisol, which are not explicitly linked to health conditions.
- Articles without an English language title and abstract.

Stage 4: Charting the data

For peer review only

A bespoke data charting tool has been developed following identification of key variables from relevant studies identified during pilot searches (see attached supplementary file for details). The data to be extracted includes information about the sample demographics (e.g., age, gender balance), type of care placement or setting, health and developmental outcomes considered, study design, and country in which the study was carried out. Bibliographic information will also be included. Data extraction from included full-text articles will be carried out by one reviewer (DRRB) and at least 20% of these extractions will be verified independently by a second reviewer.

Stage 5: Collating, summarising, and reporting the results

The results of the search and screening process will be reported using a PRISMA-ScR flow diagram.[48] The details of included studies will be presented as tables. Aggregated descriptive statistics about studies will be presented. A thematic analysis taking a descriptive-analytical stance will be carried out to identify emergent clusters of health conditions (if sufficient studies are identified). Differences in outcomes between children with and without care experience will also be summarised (if sufficient comparative studies are identified). Bibliographic information will also be collated and presented including title, author(s), year of publication, and publishing journal.

ETHICS AND DISSEMINATION

This study will review publicly-available knowledge so requires no additional ethical approval. It is the intent of the authors to present the findings of this review as an article in peer-reviewed journal and at relevant conferences. The results of this review will also inform future research into health conditions that may disproportionately affect care-experienced children, as well as potentially stimulating research into health conditions which have been under-researched in this group. This review also serves as a backdrop to a planned research project by the authors into the health and development of preschool children in

For peer review only

care in Scotland. Additionally, the findings of this review will be of interest to practitioners and policy makers working to understand and improve the health outcomes of care-experienced children. Our results will therefore also be circulated to interested stakeholders to make a direct and immediate contribution to the field.

For peer review only

Acknowledgements: DRRB would like to thank Valerie Wells (Information Scientist, MRC/CSO Social & Public Health Sciences Unit) for advice about search strategies, and Dr. Avril Mason (Consultant Paediatric Endocrinologist, NHS Greater Glasgow & Clyde) for reviewing an initial draft of the manuscript.

Contributors: DRRB was responsible for conceptualising the review, outlining the methods and search strategies, and preparation of the first draft of the manuscript. DB, MA, and ADMcM supervised the study design and gave critical input for the revision and refinement of the review methodology. All authors were significantly involved in the preparation of the final version of the manuscript and approved it for submission.

Funding: This research is funded as part of an MRC PhD studentship awarded to DRRB (MC_ST_00022). MA/DB were supported by the Economic and Social Research Council (grant number ES/T000120/1), the Medical Research Council (MC_UU_00022/2) and the Scottish Government Chief Scientist Office (SPHSU17).

Competing interests: None to declare.

Patient consent: Not required.

Provenance and peer review: Not commissioned; externally peer reviewed.

For peer review only

REFERENCES

1 Scottish Government. Children’s Social Work Statistics, 2018-19. Edinburgh: 2020. http://www.gov.scot/Resource/0049/00499452.pdf

2 Department for Education. Children looked after in England including adoptions. 2021.https://explore-education-statistics.service.gov.uk/find-statistics/children-looked-after-in-england-including-adoptions

3 Högberg U, Sennerstam R, Wester K, *et al.* Medical diagnoses among infants at entry in out-of-home care: A Swedish population-register study. *Health Science Reports* 2019;**2**:1–12. doi:10.1002/hsr2.133

4 OECD Centre on Well-being, Inclusion S and EO (WISE). *Measuring What Matters for Child Well-Being and Policies*. OECD 2021. doi:10.1787/e82fded1-en

5 Bronsard G, Alessandrini M, Fond G, *et al.* The Prevalence of Mental Disorders Among Children and Adolescents in the Child Welfare System. *Medicine* 2016;**95**:e2622. doi:10.1097/MD.0000000000002622

6 McMahon AD, Elliott L, Macpherson LMDM, *et al.* Inequalities in the dental health needs and access to dental services among looked after children in Scotland: A population data linkage study. *Archives of Disease in Childhood* 2018;**103**:39–43. doi:10.1136/archdischild-2016-312389

7 Murray ET, Lacey R, Maughan B, *et al.* Association of childhood out-of-home care status with all-cause mortality up to 42-years later: Office of National Statistics Longitudinal Study. *BMC Public Health* 2020;**20**:1–11. doi:10.1186/s12889-020-08867-3

8 Murray ET, Lacey R, Maughan B, *et al.* Non-parental care in childhood and health up to 30 years later: ONS Longitudinal Study 1971–2011. *European Journal of Public Health* 2020;**30**:1121–7. doi:10.1093/eurpub/ckaa113

9 Sacker A, Lacey RE, Maughan B, *et al.* Out-of-home care in childhood and socio-economic functioning in adulthood: ONS Longitudinal study 1971–2011. *Children and Youth Services Review* 2022;**132**:106300. doi:10.1016/j.childyouth.2021.106300

10 Engler AD, Sarpong KO, van Horne BS, *et al.* A Systematic Review of Mental Health Disorders of Children in Foster Care. *Trauma, Violence, & Abuse* 2022;**23**:255–64. doi:10.1177/1524838020941197

11 McKenna S, Donnelly M, Onyeka IN, *et al.* Experience of child welfare services and long-term adult mental health outcomes: a scoping review. *Social Psychiatry and Psychiatric Epidemiology* 2021;**56**:1115–45. doi:10.1007/s00127-021-02069-x

12 Vasileva M, Petermann F. Attachment, Development, and Mental Health in Abused and Neglected Preschool Children in Foster Care: A Meta-Analysis. *Trauma, Violence, and Abuse* 2018;**19**:443–58. doi:10.1177/1524838016669503

13 Goemans A, van Geel M, Vedder P. Over three decades of longitudinal research on the development of foster children: A meta-analysis. *Child Abuse & Neglect* 2015;**42**:121–34. doi:10.1016/j.chiabu.2015.02.003

For peer review only

14 Heady N, Watkins A, John A, *et al.* Prevalence of neurodevelopmental disorders and their impact on the health and social well-being among looked after children (LAC): a systematic review protocol. *Systematic Reviews* 2022;**11**:49. doi:10.1186/s13643-022-01923-6

15 Schneiderman JU, Leslie LK, Arnold-Clark JS, *et al.* Pediatric health assessments of young children in child welfare by placement type. *Child Abuse and Neglect* 2011;**35**:29–39. doi:10.1016/j.chiabu.2010.06.007

16 Kling S, Vinnerljung B, Hjern A. Somatic assessments of 120 Swedish children taken into care reveal large unmet health and dental care needs. *Acta Paediatrica* 2016;**105**:416–20. doi:10.1111/apa.13304

17 Turney K, Wildeman C. Mental and Physical Health of Children in Foster Care. *Pediatrics* 2016;**138**:e20161118. doi:10.1542/peds.2016-1118

18 Nathanson D, Tzioumi D. Health needs of Australian children living in out-of-home care. *Journal of Paediatrics and Child Health* 2007;**43**. doi:10.1111/j.1440-1754.2007.01193.x

19 Fleming M, McLay JS, Clark D, *et al.* Educational and health outcomes of schoolchildren in local authority care in Scotland: A retrospective record linkage study. *PLOS Medicine* 2021;**18**:e1003832. doi:10.1371/journal.pmed.1003832

20 Martin A, Ford T, Goodman R, *et al.* Physical illness in looked-after children: a cross-sectional study. *Archives of Disease in Childhood* 2014;**99**:103–7. doi:10.1136/archdischild-2013-303993

21 Verd S, Aguiló G, Kraemer M, *et al.* Past medical history: A comparison between children at the point of entry into the care system and children at home. *Journal of Child Custody* 2018;**15**:228–40. doi:10.1080/15379418.2018.1521761

22 Barsness KA, Cha E-S, Bensard DD, *et al.* The Positive Predictive Value of Rib Fractures as an Indicator of Nonaccidental Trauma in Children. *The Journal of Trauma: Injury, Infection, and Critical Care* 2003;**54**:1107–10. doi:10.1097/01.TA.0000068992.01030.A8

23 Gnanamanickam ES, Nguyen H, Armfield JM, *et al.* Hospitalizations among children involved in the child protection system: A long-term birth cohort study from infancy to adulthood using administrative data. *Child Abuse & Neglect* 2020;**107**:104518. doi:10.1016/j.chiabu.2020.104518

24 Isıyel E, Tekşam Ö, Foto-özdemir D, *et al.* Home accident or physical abuse: Evaluation of younger children presenting with trauma, burn and poisoning in the pediatric emergency department. *Turkish Journal of Pediatrics* 2018;**60**:625–32. doi:10.24953/turkjped.2018.06.002

25 Fernandez E. Reasons for Children Entering Care. In: Fernandez E, ed. *Accomplishing Permanency: Reunification Pathways and Outcomes for Foster Children*. Dordrecht: : Springer Netherlands 2013. 31–44. doi:10.1007/978-94-007-5092-0_3

26 Cusworth L, Biehal N, Whincup H, *et al.* Permanently Progressing? Children looked after away from home aged five and under in Scotland: experiences, pathways and outcomes. 2019. https://www.stir.ac.uk/media/stirling/services/research/documents/policy-

For peer review only

briefings/Children-looked-after-away-from-home-aged-five-and-under-in-Scotland-experiences,-pathways-and-outcomes.pdf

27 Mc Grath-Lone L, Dearden L, Nasim B, *et al.* Changes in first entry to out-of-home care from 1992 to 2012 among children in England. *Child Abuse and Neglect* 2016;**51**:163–71. doi:10.1016/j.chiabu.2015.10.020

28 Naughton AM, Maguire SA, Mann MK, *et al.* Emotional, Behavioral, and Developmental Features Indicative of Neglect or Emotional Abuse in Preschool Children. *JAMA Pediatrics* 2013;**167**:769. doi:10.1001/jamapediatrics.2013.192

29 Xu J, Zhu Y, Wang C, *et al.* The Dental Health of Orphan and Non-orphan Children in Fuyang City, China. *Oral Health & Preventive Dentistry* 2021;**19**:523--528. doi:10.3290/j.ohpd.b2182947

30 Simkiss DE, Stallard N, Thorogood M. A systematic literature review of the risk factors associated with children entering public care. *Child: Care, Health and Development* 2013;**39**:628–42. doi:10.1111/cch.12010

31 Reuben A, Moffitt TE, Caspi A, *et al.* Lest we forget: comparing retrospective and prospective assessments of adverse childhood experiences in the prediction of adult health. *Journal of Child Psychology and Psychiatry* 2016;**57**:1103–12. doi:10.1111/jcpp.12621

32 Bellis MA, Lowey H, Leckenby N, *et al.* Adverse childhood experiences: retrospective study to determine their impact on adult health behaviours and health outcomes in a UK population. *Journal of Public Health* 2014;**36**:81–91. doi:10.1093/pubmed/fdt038

33 Gunton KB, Wasserman BN, DeBenedictis C. Strabismus. *Primary Care: Clinics in Office Practice* 2015;**42**:393–407. doi:10.1016/j.pop.2015.05.006

34 Neil E, Gitsels L, Thoburn J. Children in care: Where do children entering care at different ages end up? An analysis of local authority administrative data. *Children and Youth Services Review* 2019;**106**:104472. doi:10.1016/j.childyouth.2019.104472

35 Lionetti F, Pastore M, Barone L. Attachment in institutionalized children: A review and meta-analysis. *Child Abuse & Neglect* 2015;**42**:135–45. doi:10.1016/j.chiabu.2015.02.013

36 Oswald SH, Heil K, Goldbeck L. History of Maltreatment and Mental Health Problems in Foster Children: A Review of the Literature. *Journal of Pediatric Psychology* 2010;**35**:462–72. doi:10.1093/jpepsy/jsp114

37 Kemmis-Riggs J, Dickes A, McAloon J. Program Components of Psychosocial Interventions in Foster and Kinship Care: A Systematic Review. *Clinical Child and Family Psychology Review* 2018;**21**:13–40. doi:10.1007/s10567-017-0247-0

38 Hickey L, Galvin K, Parolini A, *et al.* The engagement of children in out-of-home care with nursing and allied health professionals: A scoping review. *Child: Care, Health and Development* 2021;**47**:758–70. doi:10.1111/cch.12893

39 Hambrick EP, Oppenheim-Weller S, N'zi AM, *et al.* Mental health interventions for children in foster care: A systematic review. *Children and Youth Services Review* 2016;**70**:65–77. doi:10.1016/j.childyouth.2016.09.002

For peer review only

40 Marcellus L, MacKinnon K, Gordon C, *et al.* Interventions and programs that support the health and development of infants with prenatal substance exposure in foster care: a scoping review. *JBI Evidence Synthesis* 2021;**19**:1844–86. doi:10.11124/JBIES-20-00071

41 Kang-Yi CD, Adams DR. Youth with Behavioral Health Disorders Aging Out of Foster Care: a Systematic Review and Implications for Policy, Research, and Practice. *The Journal of Behavioral Health Services & Research* 2017;**44**:25–51. doi:10.1007/s11414-015-9480-9

42 Arksey H, O'Malley L. Scoping studies: towards a methodological framework. *International Journal of Social Research Methodology* 2005;**8**:19–32. doi:10.1080/1364557032000119616

43 Levac D, Colquhoun H, O'Brien KK. Scoping studies: advancing the methodology. *Implementation Science* 2010;**5**:69. doi:10.1186/1748-5908-5-69

44 Grant MJ, Booth A. A typology of reviews: an analysis of 14 review types and associated methodologies. *Health Information & Libraries Journal* 2009;**26**:91–108. doi:10.1111/j.1471-1842.2009.00848.x

45 Moffa M, Cronk R, Fejfar D, *et al.* A systematic scoping review of hygiene behaviors and environmental health conditions in institutional care settings for orphaned and abandoned children. *Science of The Total Environment* 2019;**658**:1161–74. doi:10.1016/j.scitotenv.2018.12.286

46 Sewnet SS, Derseh HA, Desyibelew HD, *et al.* Undernutrition and Associated Factors among Under-Five Orphan Children in Addis Ababa, Ethiopia, 2020: A Cross-Sectional Study. *Journal of Nutrition and Metabolism* 2021;**2021**:1–9. doi:10.1155/2021/6728497

47 Peters M, Godfrey C, McInerney P, *et al.* Chapter 11: Scoping Reviews. In: Aromataris E, Munn Z, eds. *JBI Manual for Evidence Synthesis*. JBI 2020. doi:10.46658/JBIMES-20-12

48 Tricco AC, Lillie E, Zarin W, *et al.* PRISMA Extension for Scoping Reviews (PRISMA-ScR): Checklist and Explanation. *Annals of Internal Medicine* 2018;**169**:467–73. doi:10.7326/M18-0850

49 World Health Organization. ICD-11: International classification of diseases (11th revision). 2019.https://icd.who.int/

50 Ouzzani M, Hammady H, Fedorowicz Z, *et al.* Rayyan—a web and mobile app for systematic reviews. *Systematic Reviews* 2016;**5**:210. doi:10.1186/s13643-016-0384-4

51 World Bank. World Bank Country and Lending Groups. https://datahelpdesk.worldbank.org/knowledgebase/articles/906519-world-bank-country-and-lending-groups (accessed 4 Apr 2022).

For peer review only

A. KEYWORDS

Care-experienced children

“care experienced child” OR “looked after child*” OR lac OR “out of home care” OR “foster* child*” OR “foster* care” OR “foster placement” OR “kinship care*” OR “substitute care” OR “kith and kin care” OR “friends and family care” OR “adopted child*” OR orphan* OR “residential care” OR “group home*” OR “child* home” OR “care home” OR “local authority care” OR “corporate parent” OR “child* protect*” OR “public care” OR “institution* care*” OR “supported living” OR “social care” OR “care placement” OR “child* welfare” OR “protective custody” OR “state custody”

Health

health OR healthcare OR medicat* OR condition* OR patholog* OR feeding OR growth OR thrive OR stature OR stunting OR immuni* OR infect* OR symptom* OR disease* OR illness* OR “well-being” OR wellbeing OR neurodevelop* OR treatment* OR prescri* OR hospital*

Age

infan* OR pre-school OR preschool OR baby OR neonat* OR “under 5” OR “under five” OR “early intervention” OR birth OR “young child*” OR toddler

For peer review only

B. SEARCH STRATEGY EXAMPLE

The following search strategy was designed for and used on Medline via the Ovid interface.

1 exp Child, Foster/ 163

2 exp Child, Adopted/ 142

3 exp Foster Home Care/ 3772

4 (care experienced child* or looked after child* or lac or foster* child* or adop* child* or orphan*).ab,ti. 27712

5 (("out of home" or foster* or "friends and family" or "kith and kin" or kinship or local authorit* or institution* or substitute) adj1 care).ab,ti. 9752

6 ("care home" or "group home" or "child* home").ab,ti. 3736

7 ("care placement" or "foster placement").ab,ti. 376

8 ("corporate parent" or "child* protect*" or "public care" or "protective custody" or "child* welfare" or "state custody" or "supported accomodation" or "supported living").ab,ti. 6471

9 (infan* or pre-school or preschool or baby or babies or neonat* or "under 5" or "under five" or "early intervention" or birth or "young child*" or toddler).ab,ti. 904645

10 exp Child, Preschool/ 968987

11 (health or healthcare or medicat* or condition* or patholog* or feeding or growth or thrive or stature or stunting or immuni* or infect* or symptom* or disease* or illness* or "well-being" or wellbeing or neurodevelop* or treatment* or prescri* or hospital*).ab,ti. 12478425

12 exp Child Development/ 64809

13 1 or 2 or 3 or 4 or 5 or 6 or 7 or 8 48052

14	9 or 10	1718061
15	11 or 12	12517557
16	13 and 14 and 15	5232

For peer review only

C. DATA CHARTING VARIABLES

Bibliographic variables

Article title, authors, year of publication, journal title.

Participant demographics and setting

Age (inc. measure of central tendency/dispersion), gender split, care placement type, country.

Study-related variables

Health outcomes studied, prevalence statistics/statistical methods used, effect sizes (where appropriate), study aims/objectives, study design, sample type(s), no. of care-experienced children in sample, no. of non-care children in control sample (where appropriate), key findings of relevance to review question.

For peer review only

BMJ Open

Physical health of care-experienced young children in high-income countries: a scoping review protocol

Journal:	*BMJ Open*
Manuscript ID	bmjopen-2022-063648.R1
Article Type:	Protocol
Date Submitted by the Author:	05-Aug-2022
Complete List of Authors:	Bradford, Daniel ; University of Glasgow, MRC/CSO Social and Public Health Sciences Unit<br>Allik, Mirjam; University of Glasgow, MRC/CSO Social and Public Health Sciences Unit<br>McMahon, Alex; University of Glasgow, School of Medicine, Dentistry & Nursing<br>Brown, Denise ; University of Glasgow, MRC/CSO Social and Public Health Sciences Unit
**Primary Subject Heading**:	Paediatrics
Secondary Subject Heading:	Public health
Keywords:	PAEDIATRICS, GENERAL MEDICINE (see Internal Medicine), Community child health < PAEDIATRICS

SCHOLARONE™
Manuscripts

Title: Physical health of care-experienced young children in high-income countries: a scoping review protocol

Authors: D. R. R. Bradford[1], M. Allik[1], A. D. McMahon[2], and D. Brown[1]

Affiliations:

1. MRC/CSO Social and Public Health Sciences Unit, University of Glasgow, Berkeley Square, 99 Berkeley Street, Glasgow. G3 7HR.
2. School of Medicine, Dentistry & Nursing, K12 Level 8, 378 Sauchiehall Street, Glasgow Dental Hospital & School, Glasgow. G2 3JZ.

ORCiD: D. R. R. Bradford, M. Allik, A. D. McMahon, and D. Brown

Correspondence: D. R. R. Bradford - d.bradford.1@research.gla.ac.uk

Word count: 2707 (Inc. headers, tables, in-text citations. Exc. front matter, end matter, abstract, references, supplemental file.)

Keywords: care-experienced children; child health; foster care; infant health; physical health

Contributorship statement: DRRB conceptualised the scoping review and research questions, developed the search strategy, and drafted the manuscript. DB, MA, and ADMcM critically reviewed the review methods and provided comments on all aspects of initial draft of the manuscript. All authors have made substantial intellectual contributions to the work.

For peer review only

ABSTRACT

Introduction

Care-experienced children have poorer health, developmental, and quality of life outcomes across the lifespan compared to children that are not in care. These inequities begin to manifest in the early years. The purpose of the proposed scoping review is to collate and synthesise studies of the physical health of young care-experienced children. The results of the review will help map the distribution of health outcomes, identify potential targets for intervention, and assess gaps in the literature relating to this group.

Methods and analysis

We will carry out a scoping review of the literature to identify studies of physical health outcomes in care-experienced children. Systematic literature searches will be carried out on the MEDLINE, CINAHL, and Web of Science Core Collection databases for items indexed on or before 31st August 2022. Studies will be included where the participants are aged three months or greater and less than six years. Data elements extracted from included studies will include study objectives, health outcomes, participant demographics, care setting characteristics, and bibliographic information. The results of the review will be synthesised and reported using a critical narrative approach. Comparisons between care and non-care populations will be reported if sufficient studies are identified.

Ethics and dissemination

Data will be extracted from publicly available sources so no additional ethical approval is required. Results will be published in a peer-reviewed journal article. Further, they will be shared in summary reports and presented to local authorities, care organisations, and other relevant stakeholders that can influence healthcare policy and procedure relating to young children in care.

For peer review only

Abstract word count: 250 (exc. headers)

For peer review only

STRENGTHS AND LIMITATIONS OF THIS STUDY

- The scoping review method facilitates the synthesis of evidence from varied study designs concerning physical health outcomes.
- The prospective review will follow validated methods for literature screening, data extraction, and reporting to maximise transparency and replicability.
- Exclusion of grey literature and articles without English-language abstracts are limitations of the prospective review.

For peer review only

BACKGROUND

A significant number of infants require support beyond that which their biological parents can provide. To have these additional support needs met, some children come formally under the care of social care services provided by local or national government agencies. This may mean receiving extra input from health and social care workers while remaining in the parental home; living in kinship care; living in foster care; being permanently adopted by non-biological parents; or living in residential care homes. The proportion of children in care varies internationally due to differing legal and cultural approaches. Examples from high-income countries include 14 per 1,000 children in Scotland,[1] 6.7 per 1,000 in England,[2] and 4.0 per 1,000 infants in Sweden.[3] These cross-sectional census figures underestimate the number of children that have ever been in care since there is a constant flux of children into and out of care. Although care-experienced children constitute a small portion of the population of children in high-income countries, these children deserve increased attention due to the additional challenges they face to achieving good health.[4] There is a clear motivation to provide support to improve health outcomes for such children.

Children with care experience have poorer outcomes in many domains of health and wellbeing compared to their peers in the non-care population.[5–9] Mental ill health is prevalent across the lifespan in this group and has been the subject of many reviews.[10–12] Similarly, developmental delay and disorder in socioemotional, cognitive, and behavioural domains are common in care-experienced children and these topics have been reviewed previously.[12–14] The literature on physical health and physical development problems in care-experienced children is less well-established, particularly for those in the early years. Despite physical ill health problems being highly prevalent in this population,[15–18] these health outcomes have not yet been the subject of a review. Bringing together the results of studies which investigated this topic is therefore a useful exercise and is the purpose of the present work. Our results will help identify common conditions affecting children in care,

For peer review only

highlight health conditions and inequalities that may benefit from the development of specific interventions, and identify potential gaps in the literature to be addressed.

The results of studies comparing physical health outcomes in care-experienced children and children that have never been in care are less clearly polarised than those for mental health and socioemotional, cognitive, and behavioural development, where the former group are unequivocally worse off.[17,19,20] In the physical domain there are instances where care-experienced children are observed to have a reduced disease burden, although these are typically for less severe disease. For example, asthma has been found to be less prevalent in samples of children in care than controls from the non-care population.[20,21] By contrast, Turney and Wildeman reported a significantly higher crude proportion of children aged 0—17 years in foster care with asthma.[17] However, after adjusting for birth and socioeconomic factors the difference in proportion compared to the non-care group became negligible. Fleming et al also reported no significant difference in asthma prevalence (after adjustment for birth and socioeconomic factors) in their population-level administrative health data study of school-aged children.[19] Reviewing the available literature can help to clarify such ambiguities. There is, of course, some risk that differences between care-experienced and other children in the prevalence of diagnosed health conditions are due to differences in health-seeking behaviour rather than truly better health. Regardless, improved knowledge about differences in the prevalence of physical ill health between children in care and children in the general population is needed to understand potential associations and/or causes.

While some differences in physical health conditions between children in care and children in the general population are conflicting or ambiguous, the subset of conditions caused by, or strongly associated with, abuse and neglect — such as bone fractures and poisonings — are evidently more common in care-experienced populations.[3,22–24] This is unsurprising since abuse and neglect are common reasons for a child becoming formally cared for.[25–27] Although these traumatic experiences have clear and well-documented consequences for

For peer review only

socioemotional development,[28] there are also consequences for physical health. Hospitalisations due to both injury and self-harm have been found to be more common in school-aged children in care than in their non-care peers, after adjusting for a range of socioeconomic and maternal factors.[19] Neglectful parenting also has consequences for conditions where proactive health behaviours are vital. For example, dental health problems were significantly more frequently identified in children in care during a universal dental health screening programme in Scotland.[6] This difference remained after adjustment for socioeconomic status and echoes previous studies reporting increased dental health issues in this group.[16,18,21,29] Similarly, care-experienced children are more likely to have incomplete immunisation, or to have been fully immunised at older ages.[16,18] These examples highlight that children in care face unique challenges to their health. These challenges are compounded by factors deleterious to health that are associated with poorer socioeconomic circumstances which care-experienced children more often reside in.[6,30] Identification and mitigation of the consequences of these unique risk factors can help to improve health policy and practices relating to this group.

Infancy and childhood are a period where growth and development are highly sensitive to factors in the individual's environment. Being subject to emotional and physical adversity during this period is associated with long-term negative physical health outcomes.[31,32] If untreated or uncorrected during early childhood, some malleable impairments to health may become permanent. Strabismus (misalignment of the visual axes of the eyes) is an example of such a correctable condition. Intervention to treat this condition in the early years is key to avoiding this becoming a lifelong issue.[33] Additionally, children that enter care early in life also tend to experience different pathways to those becoming cared for during middle childhood or adolescence.[34] Taken together, this suggests children with experience of formal care in the early years may exhibit a distinct cluster of healthcare needs compared to similarly-aged children that are not in care. Furthermore, their healthcare needs likely differ from children that first become cared for later in life. By investigating physical health in the

For peer review only

younger care-experienced group in more detail it is possible that opportunities for early intervention can be better exploited to improve subsequent wellbeing.

Study objectives

Previous reviews and meta-analyses have looked at socioemotional and behavioural development,[12,13,28,35] mental health,[10,12,36] effectiveness of interventions and engagement with healthcare providers,[37–39] or focused on specific subgroups of children with care-experience (e.g., children with prenatal substance exposure,[40] young adults leaving care[41]). Based on preliminary searches of MEDLINE and the Cochrane Database of Systematic Reviews, it appears there has not been a review of physical health outcomes in young care-experienced children to date. The present review aims to fill that gap. A scoping review is an appropriate methodology for this purpose. This type of review will establish the scope and common themes within existing research into the topic and indicate which physical health conditions are frequently observed in young care-experienced children. This will be of interest to policy makers and healthcare practitioners.[42–44] Further, under-researched health conditions may be identified by comparing the results of this review with the more substantial body of literature on the physical health of young children that are not in care.

Concept definition

This review will address the literature on physical health. We have defined physical health conditions as those primarily rooted or expressed in physiology. While we recognise that psychiatric conditions and socioemotional, cognitive, and behavioural developmental conditions can have organic and/or physiological aspects, these conditions have been previously reviewed (see above). In addition, the proposed review is restricted to children in care in high-income countries. This is because these children face notably different types of physical health conditions to those in low- and middle-income countries.[45,46]

For peer review only

METHODS AND ANALYSIS

This review has been designed in line with the Joanna Briggs Institute (JBI) guidelines for carrying out scoping reviews.[47] It is also informed by Arksey and O'Malley's original framework [42] and the additional guidance laid out by Levac, Colquhoun, and O'Brien.[43] The five steps of the review procedure of Arksey and O'Malley are detailed below. The JBI framework aligns with the Preferred Reporting Items for Systematic Reviews and Meta-analyses extension (PRISMA-ScR) statement on reporting and conduct of scoping reviews,[48] also used herein.

Patient and public involvement

There was no patient nor public involvement in this review protocol.

Stage 1: Identifying the research question

The Population, Concept, Context framework (PCC) was used to define the review question. Elements of the PCC are described in detail in in Table 1. This review intends to primarily answer the question: "Which physical health conditions have been studied or observed to affect young care-experienced children in high-income countries?" If there are sufficient relevant primary studies which compare care-experienced children to control groups in the general, non-care population then the review will also address the question: "How do these health conditions affect children in care compared to their peers in the general population?"

Population	Care-experienced children who meet all following criteria:<br>• Aged greater than or equal to three months and less than 6y<br>• Have experience of formal care settings listed in the introduction of this protocol, or similar settings<br>• Not adopted during the first three months of their life<br>• Not cared for in healthcare facilities, to avoid confounding

For peer review only

Concept	Physical health outcomes will be considered as those health conditions which are primarily rooted in or expressed through physiology. As a guide, these conditions will be those that are not listed in the International Classification of Diseases (11th Revision) [49] diagnostic manual top-level entity on *Mental, behavioural or neurodevelopmental disorders*.
Context	Included studies will be restricted to those carried out in high-income countries. This is because children in care in low- and middle-income countries face significantly different physical health issues.[45,46]

Table 1: Detailed description of the Population, Concept, and Context used a foundation for the review research questions.

Stage 2: Identifying relevant studies

Keywords and synonyms to identify 1) care-experienced children; 2) health and wellbeing; and 3) children aged greater than or equal to three months and less than six years will be combined to search the bibliographic databases. Studies of the health of care-experienced children previously known to the authors were used to identify keywords to form the basis of the search strategy for this review. Iterative pilot searches of MEDLINE were carried out to identify additional relevant keywords. This process ended when saturation was reached, and additional keywords/variants did not bring forth additional search results. Advice on keywords and search strategy was also taken from an Information Scientist within the University of Glasgow MRC/CSO Social and Public Health Sciences Unit. These keywords are detailed in an attached supplementary file. The review will search for relevant studies indexed by the MEDLINE, CINAHL, and Web of Science Core Collection (Science Citation Index [SCI-EXPANDED] and Social Sciences Citation Index [SSCI]). An example search strategy for MEDLINE (via the Ovid interface) is provided in an attached supplementary file. The search strategy will be adapted appropriately for the remaining databases. Following searches, all results will be imported to Rayyan.[50] Duplicates will be removed.

For peer review only

Stage 3: Study selection

Search results will be screened for relevance to the Population Concept, and Context described above. More specific inclusion and exclusion criteria are described below. Screening will be carried out using Rayyan.[50] Screening will take place in two stages: title and abstract screening then full texts. All titles and abstracts will be screened by DRRB and a minimum of 20% will be screened independently by a second reviewer. If it is unclear whether an article should be included based on the details in the title/abstract and there is potential for it to be relevant then it will be progressed for full-text screening (e.g., if the study clearly includes care-experienced children but the age of participants is not immediately apparent it will be progressed to the full-text review stage). Non-English-language articles with English-language abstracts that indicate relevance to this review will be translated and included. Crude inter-rater agreement will be reported along with Cohen's Kappa and Scott's Pi. If crude agreement falls below 95% in this sample of 20% of titles/abstracts then all items will be screened by at least two reviewers. During the next stage of screening all full-texts will be screened by DRRB and at least 20% will be screened by a second reviewer. Crude inter-rater agreement will be reported along with Cohen's Kappa [51] and Scott's Pi.[52] If crude agreement falls below 95% then all full-texts will be screened by at least two reviewers. Disagreements at any stage of screening will be resolved by discussion between the two reviewers. If consensus is not achieved, a third reviewer will make the final decision. Reasons for exclusion will be recorded and documented at each stage and reported in line as per the PRISMA-ScR guidelines.[48]

Inclusion criteria

- Included studies will consider children currently living in family care with additional formal support from social or child protection services, kinship care, foster care, residential care, as well as children living with adopted families. Studies of children

with previous experience of these settings will also be included, as will studies which aggregate groups of children either currently in or having previously been in care.

- Studies will be included if all children are aged greater than or equal to three months and less than six years, or if subgroup data for this group are reported.
- Study outcomes must include physical health conditions or diagnoses by a health professional, diagnostic manual, or validated measure(s). Studies which have made minor modifications to validated instruments may be included at reviewers' discretion. Studies which use parental report of a current or previous diagnosis by a clinician will be included.
- Studies will be included if they were carried out in high-income countries as defined by the World Bank.[53]
- Only articles and reports published in peer-reviewed journals will be included.

Exclusion criteria

- Studies of children living in healthcare settings will be excluded to avoid confounding.
- Papers that look at only physiological markers will be excluded. For example, studies of expression of hormones such as salivary cortisol, will be excluded unless explicitly linked to a health condition.
- Articles without an English language title and abstract.

Stage 4: Charting the data

A bespoke data charting tool has been developed following identification of key variables from relevant studies identified during pilot searches (see attached supplementary file for details). The data to be extracted includes information about the sample demographics (e.g., age, gender balance), type of care placement or setting, health and developmental outcomes considered, study design, and country in which the study was carried out. Bibliographic information will also be included. Data extraction from included full-text articles

For peer review only

will be carried out by one reviewer (DRRB) and at least 20% of these extractions will be verified independently by a second reviewer.

Stage 5: Collating, summarising, and reporting the results

The results of the search and screening process will be reported using a PRISMA-ScR flow diagram.[48] The details of included studies will be presented as tables. Aggregated descriptive statistics about studies will be presented. A thematic analysis will be carried out to identify emergent clusters of health conditions (if sufficient studies are identified). Differences in outcomes between children with and without care experience will also be summarised (if sufficient comparative studies are identified). Bibliographic information will also be collated and presented including title, author(s), year of publication, and publishing journal.

ETHICS AND DISSEMINATION

This study will review publicly-available knowledge so requires no additional ethical approval. It is the intent of the authors to present the findings of this review as an article in a peer-reviewed journal and at relevant conferences. The results of this review will also inform future research into health conditions that may disproportionately affect care-experienced children, as well as potentially stimulating research into health conditions which have been under-researched in this group. This review also serves as a backdrop to a planned research project by the authors into the health and development of preschool children in care in Scotland. Additionally, the findings of this review will be of interest to practitioners and policy makers working to understand and improve the health outcomes of care-experienced children. Our results will therefore also be circulated to interested stakeholders to make a direct and immediate contribution to the field.

For peer review only

Acknowledgements: DRRB would like to thank Valerie Wells (Information Scientist, MRC/CSO Social & Public Health Sciences Unit) for advice about search strategies, and Dr. Avril Mason (Consultant Paediatric Endocrinologist, NHS Greater Glasgow & Clyde) for reviewing an initial draft of the manuscript.

Contributors: DRRB was responsible for conceptualising the review, outlining the methods and search strategies, and preparation of the first draft of the manuscript. DB, MA, and ADMcM supervised the study design and gave critical input for the revision and refinement of the review methodology. All authors were significantly involved in the preparation of the final version of the manuscript and approved it for submission.

Funding: This research is funded as part of an MRC PhD studentship awarded to DRRB (MC_ST_00022). MA/DB were supported by the Economic and Social Research Council (grant number ES/T000120/1), the Medical Research Council (MC_UU_00022/2) and the Scottish Government Chief Scientist Office (SPHSU17).

Competing interests: None to declare.

Patient consent: Not required.

Provenance and peer review: Not commissioned; externally peer reviewed.

For peer review only

REFERENCES

1 Scottish Government. Children's Social Work Statistics, 2018-19. Edinburgh: 2020. http://www.gov.scot/Resource/0049/00499452.pdf

2 Department for Education. Children looked after in England including adoptions. 2021.https://explore-education-statistics.service.gov.uk/find-statistics/children-looked-after-in-england-including-adoptions

3 Högberg U, Sennerstam R, Wester K, *et al.* Medical diagnoses among infants at entry in out-of-home care: A Swedish population-register study. *Health Science Reports* 2019;**2**:1–12. doi:10.1002/hsr2.133

4 OECD Centre on Well-being, Inclusion S and EO (WISE). *Measuring What Matters for Child Well-Being and Policies*. OECD 2021. doi:10.1787/e82fded1-en

5 Bronsard G, Alessandrini M, Fond G, *et al.* The Prevalence of Mental Disorders Among Children and Adolescents in the Child Welfare System. *Medicine* 2016;**95**:e2622. doi:10.1097/MD.0000000000002622

6 McMahon AD, Elliott L, Macpherson LMDM, *et al.* Inequalities in the dental health needs and access to dental services among looked after children in Scotland: A population data linkage study. *Archives of Disease in Childhood* 2018;**103**:39–43. doi:10.1136/archdischild-2016-312389

7 Murray ET, Lacey R, Maughan B, *et al.* Association of childhood out-of-home care status with all-cause mortality up to 42-years later: Office of National Statistics Longitudinal Study. *BMC Public Health* 2020;**20**:1–11. doi:10.1186/s12889-020-08867-3

8 Murray ET, Lacey R, Maughan B, *et al.* Non-parental care in childhood and health up to 30 years later: ONS Longitudinal Study 1971–2011. *European Journal of Public Health* 2020;**30**:1121–7. doi:10.1093/eurpub/ckaa113

9 Sacker A, Lacey RE, Maughan B, *et al.* Out-of-home care in childhood and socio-economic functioning in adulthood: ONS Longitudinal study 1971–2011. *Children and Youth Services Review* 2022;**132**:106300. doi:10.1016/j.childyouth.2021.106300

10 Engler AD, Sarpong KO, van Horne BS, *et al.* A Systematic Review of Mental Health Disorders of Children in Foster Care. *Trauma, Violence, & Abuse* 2022;**23**:255–64. doi:10.1177/1524838020941197

11 McKenna S, Donnelly M, Onyeka IN, *et al.* Experience of child welfare services and long-term adult mental health outcomes: a scoping review. *Social Psychiatry and Psychiatric Epidemiology* 2021;**56**:1115–45. doi:10.1007/s00127-021-02069-x

12 Vasileva M, Petermann F. Attachment, Development, and Mental Health in Abused and Neglected Preschool Children in Foster Care: A Meta-Analysis. *Trauma, Violence, and Abuse* 2018;**19**:443–58. doi:10.1177/1524838016669503

13 Goemans A, van Geel M, Vedder P. Over three decades of longitudinal research on the development of foster children: A meta-analysis. *Child Abuse & Neglect* 2015;**42**:121–34. doi:10.1016/j.chiabu.2015.02.003

For peer review only

14 Heady N, Watkins A, John A, *et al.* Prevalence of neurodevelopmental disorders and their impact on the health and social well-being among looked after children (LAC): a systematic review protocol. *Systematic Reviews* 2022;**11**:49. doi:10.1186/s13643-022-01923-6

15 Schneiderman JU, Leslie LK, Arnold-Clark JS, *et al.* Pediatric health assessments of young children in child welfare by placement type. *Child Abuse and Neglect* 2011;**35**:29–39. doi:10.1016/j.chiabu.2010.06.007

16 Kling S, Vinnerljung B, Hjern A. Somatic assessments of 120 Swedish children taken into care reveal large unmet health and dental care needs. *Acta Paediatrica* 2016;**105**:416–20. doi:10.1111/apa.13304

17 Turney K, Wildeman C. Mental and Physical Health of Children in Foster Care. *Pediatrics* 2016;**138**:e20161118. doi:10.1542/peds.2016-1118

18 Nathanson D, Tzioumi D. Health needs of Australian children living in out-of-home care. *Journal of Paediatrics and Child Health* 2007;**43**. doi:10.1111/j.1440-1754.2007.01193.x

19 Fleming M, McLay JS, Clark D, *et al.* Educational and health outcomes of schoolchildren in local authority care in Scotland: A retrospective record linkage study. *PLOS Medicine* 2021;**18**:e1003832. doi:10.1371/journal.pmed.1003832

20 Martin A, Ford T, Goodman R, *et al.* Physical illness in looked-after children: a cross-sectional study. *Archives of Disease in Childhood* 2014;**99**:103–7. doi:10.1136/archdischild-2013-303993

21 Verd S, Aguiló G, Kraemer M, *et al.* Past medical history: A comparison between children at the point of entry into the care system and children at home. *Journal of Child Custody* 2018;**15**:228–40. doi:10.1080/15379418.2018.1521761

22 Barsness KA, Cha E-S, Bensard DD, *et al.* The Positive Predictive Value of Rib Fractures as an Indicator of Nonaccidental Trauma in Children. *The Journal of Trauma: Injury, Infection, and Critical Care* 2003;**54**:1107–10. doi:10.1097/01.TA.0000068992.01030.A8

23 Gnanamanickam ES, Nguyen H, Armfield JM, *et al.* Hospitalizations among children involved in the child protection system: A long-term birth cohort study from infancy to adulthood using administrative data. *Child Abuse & Neglect* 2020;**107**:104518. doi:10.1016/j.chiabu.2020.104518

24 Isıyel E, Tekşam Ö, Foto-özdemir D, *et al.* Home accident or physical abuse: Evaluation of younger children presenting with trauma, burn and poisoning in the pediatric emergency department. *Turkish Journal of Pediatrics* 2018;**60**:625–32. doi:10.24953/turkjped.2018.06.002

25 Fernandez E. Reasons for Children Entering Care. In: Fernandez E, ed. *Accomplishing Permanency: Reunification Pathways and Outcomes for Foster Children*. Dordrecht: : Springer Netherlands 2013. 31–44. doi:10.1007/978-94-007-5092-0_3

26 Cusworth L, Biehal N, Whincup H, *et al.* Permanently Progressing? Children looked after away from home aged five and under in Scotland: experiences, pathways and outcomes. 2019. https://www.stir.ac.uk/media/stirling/services/research/documents/policy-

For peer review only

briefings/Children-looked-after-away-from-home-aged-five-and-under-in-Scotland-experiences,-pathways-and-outcomes.pdf

27 Mc Grath-Lone L, Dearden L, Nasim B, *et al.* Changes in first entry to out-of-home care from 1992 to 2012 among children in England. *Child Abuse and Neglect* 2016;**51**:163–71. doi:10.1016/j.chiabu.2015.10.020

28 Naughton AM, Maguire SA, Mann MK, *et al.* Emotional, Behavioral, and Developmental Features Indicative of Neglect or Emotional Abuse in Preschool Children. *JAMA Pediatrics* 2013;**167**:769. doi:10.1001/jamapediatrics.2013.192

29 Xu J, Zhu Y, Wang C, *et al.* The Dental Health of Orphan and Non-orphan Children in Fuyang City, China. *Oral Health & Preventive Dentistry* 2021;**19**:523--528. doi:10.3290/j.ohpd.b2182947

30 Simkiss DE, Stallard N, Thorogood M. A systematic literature review of the risk factors associated with children entering public care. *Child: Care, Health and Development* 2013;**39**:628–42. doi:10.1111/cch.12010

31 Reuben A, Moffitt TE, Caspi A, *et al.* Lest we forget: comparing retrospective and prospective assessments of adverse childhood experiences in the prediction of adult health. *Journal of Child Psychology and Psychiatry* 2016;**57**:1103–12. doi:10.1111/jcpp.12621

32 Bellis MA, Lowey H, Leckenby N, *et al.* Adverse childhood experiences: retrospective study to determine their impact on adult health behaviours and health outcomes in a UK population. *Journal of Public Health* 2014;**36**:81–91. doi:10.1093/pubmed/fdt038

33 Gunton KB, Wasserman BN, DeBenedictis C. Strabismus. *Primary Care: Clinics in Office Practice* 2015;**42**:393–407. doi:10.1016/j.pop.2015.05.006

34 Neil E, Gitsels L, Thoburn J. Children in care: Where do children entering care at different ages end up? An analysis of local authority administrative data. *Children and Youth Services Review* 2019;**106**:104472. doi:10.1016/j.childyouth.2019.104472

35 Lionetti F, Pastore M, Barone L. Attachment in institutionalized children: A review and meta-analysis. *Child Abuse & Neglect* 2015;**42**:135–45. doi:10.1016/j.chiabu.2015.02.013

36 Oswald SH, Heil K, Goldbeck L. History of Maltreatment and Mental Health Problems in Foster Children: A Review of the Literature. *Journal of Pediatric Psychology* 2010;**35**:462–72. doi:10.1093/jpepsy/jsp114

37 Kemmis-Riggs J, Dickes A, McAloon J. Program Components of Psychosocial Interventions in Foster and Kinship Care: A Systematic Review. *Clinical Child and Family Psychology Review* 2018;**21**:13–40. doi:10.1007/s10567-017-0247-0

38 Hickey L, Galvin K, Parolini A, *et al.* The engagement of children in out-of-home care with nursing and allied health professionals: A scoping review. *Child: Care, Health and Development* 2021;**47**:758–70. doi:10.1111/cch.12893

39 Hambrick EP, Oppenheim-Weller S, N'zi AM, *et al.* Mental health interventions for children in foster care: A systematic review. *Children and Youth Services Review* 2016;**70**:65–77. doi:10.1016/j.childyouth.2016.09.002

For peer review only

40 Marcellus L, MacKinnon K, Gordon C, *et al.* Interventions and programs that support the health and development of infants with prenatal substance exposure in foster care: a scoping review. *JBI Evidence Synthesis* 2021;**19**:1844–86. doi:10.11124/JBIES-20-00071

41 Kang-Yi CD, Adams DR. Youth with Behavioral Health Disorders Aging Out of Foster Care: a Systematic Review and Implications for Policy, Research, and Practice. *The Journal of Behavioral Health Services & Research* 2017;**44**:25–51. doi:10.1007/s11414-015-9480-9

42 Arksey H, O'Malley L. Scoping studies: towards a methodological framework. *International Journal of Social Research Methodology* 2005;**8**:19–32. doi:10.1080/1364557032000119616

43 Levac D, Colquhoun H, O'Brien KK. Scoping studies: advancing the methodology. *Implementation Science* 2010;**5**:69. doi:10.1186/1748-5908-5-69

44 Grant MJ, Booth A. A typology of reviews: an analysis of 14 review types and associated methodologies. *Health Information & Libraries Journal* 2009;**26**:91–108. doi:10.1111/j.1471-1842.2009.00848.x

45 Moffa M, Cronk R, Fejfar D, *et al.* A systematic scoping review of hygiene behaviors and environmental health conditions in institutional care settings for orphaned and abandoned children. *Science of The Total Environment* 2019;**658**:1161–74. doi:10.1016/j.scitotenv.2018.12.286

46 Sewnet SS, Derseh HA, Desyibelew HD, *et al.* Undernutrition and Associated Factors among Under-Five Orphan Children in Addis Ababa, Ethiopia, 2020: A Cross-Sectional Study. *Journal of Nutrition and Metabolism* 2021;**2021**:1–9. doi:10.1155/2021/6728497

47 Peters M, Godfrey C, McInerney P, *et al.* Chapter 11: Scoping Reviews. In: Aromataris E, Munn Z, eds. *JBI Manual for Evidence Synthesis*. JBI 2020. doi:10.46658/JBIMES-20-12

48 Tricco AC, Lillie E, Zarin W, *et al.* PRISMA Extension for Scoping Reviews (PRISMA-ScR): Checklist and Explanation. *Annals of Internal Medicine* 2018;**169**:467–73. doi:10.7326/M18-0850

49 World Health Organization. ICD-11: International classification of diseases (11th revision). 2019.https://icd.who.int/

50 Ouzzani M, Hammady H, Fedorowicz Z, *et al.* Rayyan—a web and mobile app for systematic reviews. *Systematic Reviews* 2016;**5**:210. doi:10.1186/s13643-016-0384-4

51 Cohen J. A Coefficient of Agreement for Nominal Scales. *Educational and Psychological Measurement* 1960;**20**:37–46. doi:10.1177/001316446002000104

52 Scott WA. Reliability of Content Analysis: The Case of Nominal Scale Coding. *Public Opinion Quarterly* 1955;**19**:321. doi:10.1086/266577

53 World Bank. World Bank Country and Lending Groups. https://datahelpdesk.worldbank.org/knowledgebase/articles/906519-world-bank-country-and-lending-groups (accessed 4 Apr 2022).

A. KEYWORDS

Care-experienced children

“care experienced child” OR cec OR “looked after child*” OR lac OR lacyp OR “out of home care” OR “foster* child*” OR “foster* care” OR “foster placement” OR “kinship care*” OR “substitute care” OR “kith and kin care” OR “friends and family care” OR “adopted child*” OR orphan* OR “residential care” OR “group home*” OR “child* home” OR “care home” OR “local authority care” OR “corporate parent” OR “child* protect*” OR “public care” OR “institution* care*” OR “supported living” OR “social care” OR “care placement” OR “child* welfare” OR “protective custody” OR “state custody”

Health

health OR healthcare OR medicat* OR condition* OR patholog* OR feeding OR growth OR thriv* OR stature OR stunting OR immuni* OR infect* OR symptom* OR disease* OR illness* OR “well-being” OR wellbeing OR neurodevelop* OR treatment* OR prescri* OR hospital*

Age

infan* OR pre-school OR preschool OR baby OR neonat* OR “under 6” OR “under six” OR “early intervention” OR birth OR “young child*” OR toddler

B. SEARCH STRATEGIES

MEDLINE via Ovid

Note: Lines beginning with “exp” instruct the database to explode the subject heading to include subsidiary terms.

1	exp Child, Foster/
2	exp Child, Adopted/
3	exp Foster Home Care/
4	(“care experienced child*” OR “looked after child*” OR lac OR “foster* child*” OR “adop* child*” OR orphan*).ab,ti.
5	(("out of home" OR foster* OR "friends and family" OR "kith and kin" OR kinship OR local authorit* OR institution* OR substitute) adj1 care).ab,ti.
6	("care home" OR "group home" OR "child* home").ab,ti.
7	("care placement" OR "foster placement").ab,ti.
8	("corporate parent" OR "child* protect*" OR "public care" OR "protective custody" OR "child* welfare" OR "state custody" OR "supported accommodation" OR "supported living").ab,ti.
9	(infan* OR pre-school OR preschool OR baby OR babies OR neonat* OR "under 6" OR "under six" OR "early intervention" OR birth OR "young child*" OR toddler*).ab,ti.
10	exp Child, Preschool/
11	(health OR healthcare OR medicat* OR condition* OR patholog* OR feeding OR growth OR thriv* OR stature OR stunting OR immuni* OR infect* OR symptom* OR disease* OR illness* OR "well-being" OR wellbeing OR neurodevelop* OR treatment* OR prescri* OR hospital*).ab,ti.
12	exp Child Development/
13	1 OR 2 OR 3 OR 4 OR 5 OR 6 OR 7 OR 8
14	9 OR 10
15	11 OR 12
16	13 AND 14 AND 15

For peer review only

CINAHL via EBSCOHost

Note: Lines beginning with MH are subject headings/keywords. The “+” operator instructs the database to explode the subject heading to include subsidiary terms.

S1	MH(Child, Abandoned OR Child, Adopted OR Child, Foster OR Child, Institutionalized OR Foster Home Care)
S2	TX(care experienced child* OR looked after child* OR lac OR foster* child* OR adop* child* OR orphan*)
S3	TX(("out of home" OR foster* OR "friends and family" OR "kith and kin" OR kinship OR local authorit* OR institution* OR substitute) N1 care)
S4	TX("care home" OR "group home" OR "child* home")
S5	TX("care placement" OR "foster placement")
S6	TX("corporate parent" OR "child* protect*" OR "public care" OR "protective custody" OR "child* welfare" OR "state custody" OR "supported accommodation" OR "supported living")
S7	MH(“Infant+” OR Child, Preschool)
S8	TX(infan* OR pre-school OR preschool OR baby OR babies OR neonat* OR "under 6" OR "under six" OR "early intervention" OR birth OR "young child*" OR toddler*)
S9	MH(Child Health OR Dental Care for Children OR “Child Health Services+”)
S10	TX(health OR healthcare OR medicat* OR condition* OR patholog* OR feeding OR growth OR thriv* OR stature OR stunting OR immuni* OR infect* OR symptom* OR disease* OR illness* OR "well-being" OR wellbeing OR neurodevelop* OR treatment* OR prescri* OR hospital*)
S11	S1 OR S2 OR S3 OR S4 OR S5 OR S6
S12	S7 OR S8
S13	S9 OR S10
S14	S11 AND S12 AND S13

For peer review only

Web of Science Core Collection

1 TI=("care experienced child*" OR "looked after child*" OR lac OR "foster* child*" OR "adop* child*" OR orphan*) OR AB=("care experienced child*" OR "looked after child*" OR lac OR "foster* child*" OR "adop* child*" OR orphan*)

2 TI=(("out of home" OR foster* OR "friends and family" OR "kith and kin" OR kinship OR “local authorit*” OR institution* OR substitute) NEAR/1 care) OR AB=(("out of home" OR foster* OR "friends and family" OR "kith and kin" OR kinship OR “local authorit*” OR institution* OR substitute) NEAR/1 care)

3 TI=("care home" OR "group home" OR "child* home") OR AB=("care home" OR "group home" OR "child* home")

4 TI=("care placement" OR "foster placement") OR AB=("care placement" OR "foster placement")

5 TI=("corporate parent" OR "child* protect*" OR "public care" OR "protective custody" OR "child* welfare" OR "state custody" OR "supported accommodation" OR "supported living") OR AB=("corporate parent" OR "child* protect*" OR "public care" OR "protective custody" OR "child* welfare" OR "state custody" OR "supported accommodation" OR "supported living")

6 TI=(infan* OR pre-school OR preschool OR baby OR babies OR neonat* OR "under 6" OR "under six" OR "early intervention" OR birth OR "young child*" OR toddler*) OR AB=(infan* OR pre-school OR preschool OR baby OR babies OR neonat* OR "under 6" OR "under six" OR "early intervention" OR birth OR "young child*" OR toddler*)

7 TI=(health OR healthcare OR medicat* OR condition* OR patholog* OR feeding OR growth OR thriv* OR stature OR stunting OR immuni* OR infect* OR symptom* OR disease* OR illness* OR "well-being" OR wellbeing OR neurodevelop* OR treatment* OR prescri* OR hospital*) OR AB=(health OR healthcare OR medicat* OR condition* OR patholog* OR feeding OR growth OR thriv* OR stature OR stunting OR immuni* OR infect* OR symptom* OR disease* OR illness* OR "well-being" OR wellbeing OR neurodevelop* OR treatment* OR prescri* OR hospital*)

8 1 OR 2 OR 3 OR 4 OR 5

9 6 AND 7 AND 8

For peer review only

C. DATA CHARTING VARIABLES

Bibliographic variables

Article title, authors, year of publication, journal title.

Participant demographics and setting

Age (inc. measure of central tendency/dispersion), gender split, care placement type, country.

Study-related variables

Health outcomes studied, prevalence statistics/statistical methods used, effect sizes (where appropriate), study aims/objectives, study design, sample type(s), no. of care-experienced children in sample, no. of non-care children in control sample (where appropriate), key findings of relevance to review question.

For peer review only
